# A Case of Snake Bite Successfully Treated with Calomel and Iodide of Potassium

**Published:** 1864-12

**Authors:** J. J. Knott

**Affiliations:** Assistant-Surgeon Fifty-Third Georgia Regiment.


ART. V.—*A Case of Snake-Bite successfully treated with Calomel and Iodide of Potassium.* By J. J. KNOTT, Assistant-Surgeon Fifty-Third Georgia Regiment.

Thomas Williamson, private company "I," Fifty-Third Georgia regiment, was admitted into Infirmary on the night of the fourth of July, having been bitten by a serpent known as the CEUCHRIS or moccasin. I found, on examination, the left hand and arm for some distance above the elbow much swollen, attended with severe pain of this member. I ordered a poultice of carb. ammonia and flax seed, to be applied over the wound.

July 5—Examined the arm and found that the swelling had increased considerably and pain augmented, the patient complaining of nausea, severe pain in head, with a sense of dizziness. Having no preparation of arsenic at hand, I determined to try a new mode of treatment, viz: the *anti syphilitic*, regarding the indication to be filled as the same. I therefore ordered No. 1, hydrarg. chloridi mitis, eighteen grains; pulv. opii, three grains; fiat pulv. No. 6, one to be given every four hours—No. 2, Iodide potassium, two ounces; aqua fontana, two drachms; teaspoonful every four hours.

July 6—Symptoms somewhat more unfavorable; treatment continued.

July 7—Symptoms more favorable; swelling subsiding. From this time the patient continued to improve, and at the present time the swelling has entirely subsided, as well as other symptoms.

I have thus briefly reported this case, which you are at liberty to publish, should you deem it worthy of a place in your columns. I can not help but regard these remedies as being fully as efficacious as "Bibrons Antidote." The articles are more accessible than those contained in Bibrons, their exhibition more harmless, and their *modus operandi* the same.

